# This *Déjà Vu* Feeling—Analysis of Multidomain Protein Evolution in Eukaryotic Genomes

**DOI:** 10.1371/journal.pcbi.1002701

**Published:** 2012-11-15

**Authors:** Christian M. Zmasek, Adam Godzik

**Affiliations:** Program in Bioinformatics and Systems Biology, Sanford-Burnham Medical Research Institute, La Jolla, California, United States of America; University College London, United Kingdom

## Abstract

Evolutionary innovation in eukaryotes and especially animals is at least partially driven by genome rearrangements and the resulting emergence of proteins with new domain combinations, and thus potentially novel functionality. Given the random nature of such rearrangements, one could expect that proteins with particularly useful multidomain combinations may have been rediscovered multiple times by parallel evolution. However, existing reports suggest a minimal role of this phenomenon in the overall evolution of eukaryotic proteomes. We assembled a collection of 172 complete eukaryotic genomes that is not only the largest, but also the most phylogenetically complete set of genomes analyzed so far. By employing a maximum parsimony approach to compare repertoires of Pfam domains and their combinations, we show that independent evolution of domain combinations is significantly more prevalent than previously thought. Our results indicate that about 25% of all currently observed domain combinations have evolved multiple times. Interestingly, this percentage is even higher for sets of domain combinations in individual species, with, for instance, 70% of the domain combinations found in the human genome having evolved independently at least once in other species. We also show that previous, much lower estimates of this rate are most likely due to the small number and biased phylogenetic distribution of the genomes analyzed. The process of independent emergence of identical domain combination is widespread, not limited to domains with specific functional categories. Besides data from large-scale analyses, we also present individual examples of independent domain combination evolution. The surprisingly large contribution of parallel evolution to the development of the domain combination repertoire in extant genomes has profound consequences for our understanding of the evolution of pathways and cellular processes in eukaryotes and for comparative functional genomics.

## Author Summary

Most proteins in eukaryotes are composed of two or more domains, evolutionary independent units with (often) their own individual functions. The specific repertoire of multidomain proteins in a given species defines the topology of pathways and networks that carry out its metabolic and regulatory processes. When proteins with new domain combinations emerge by gene fusion and fission, it directly affects topology of cellular networks in this organism. To better understand the evolution of such networks we analyzed a large set of eukaryotic genomes for the evolutionary history of known domain combinations. Our analysis shows that 70% of all domain combinations present in the human genome independently appeared in at least one other eukaryotic genome. Overall, over 25% of all known multidomain architectures emerged independently several times in the history of life. The difference between a global and species specific picture can be explained by the existence of a core set of domain combinations that keeps reemerging in different species, which are accompanied by a smaller number of unique domain combinations that do not appear anywhere else.

## Introduction

Most eukaryotic proteins are composed of multiple domains, units with their own evolutionary history and, often, specific and conserved functions. The ordered arrangement of all domains in a given protein constitutes its architecture. Protein architecture can also be described in a simplified way as a list of binary domain combinations. While not completely equivalent, both views provide similar insights, and in this manuscript we will predominantly use the latter. Many domains can combine with different partner domains and, as a result, form a wide variety of domain combinations, often even within the same species [1]. Bringing together multiple domains in one protein creates a distinct entity, combining functions of its constituents. The emergence of proteins with new domain combinations is thought to be a major mechanism of evolution of new functionality in eukaryotic genomes [2], [3]. It is especially important in the evolution of pathways, where physical proximity of domains in multidomain proteins links different elements of the pathway; thus, emergence of a new domain combination may rearrange pathways or processes in the cell [3]. The modular structure of eukaryotic proteins provides a mechanism that promotes differentiation and variation of protein functions despite the existence of only a limited number of domains. One can argue that the ease with which new domain combinations can be created provides eukaryotic genomes with the flexibility/plasticity that horizontal gene transfer and mobile genomic elements bring to prokaryotic genomes.

The domain repertoires of most eukaryotes are remarkably similar, both in size as well as in their content [4], [5]. In contrast, the number of distinct domain combinations found in multidomain proteins shows more variability between different organisms and correlates strongly with organism complexity and lifestyle [6], [7]. To some extent, the evolution of eukaryotes and major milestones in their phylogeny, leading to the rise of complex organisms, such as humans, can be linked to the emergence of specific protein architectures rather than to the emergence of new domains. This finding has prompted many studies on the evolution of multidomain proteins, especially those that are involved in regulation of pathways such as apoptosis or innate immunity [5], [8]. Special attention was, for instance, put on “promiscuous” domains—domains that have been found in combination with a particularly large number of partner domains [9]–[11]. Many such domains, for instance the PDZ or SH3 domains, are protein–protein interaction domains that “recruit” proteins to specific signaling pathways. Several groups attempted to infer domain architectures in ancestral genomes to be able to better study the evolution of new functionalities and processes carried out by such proteins 12–14. The dynamics of domain rearrangements add an additional dimension to the analysis of the evolution of gene families, which, on top of point mutations, deletions, and insertions, can also include gains and losses of entire domains. Proteins can gain (or lose) new domains in genome rearrangements, creating (or removing) domain combinations [15], [16]. In particular, the emergence of animals and, even more so, the emergence of vertebrates, have been associated with the appearance of novel domain combinations [2], [17]. It has been suggested that new domain combinations allowed for functional diversification and contributed decisively to the evolution of complex multicellular systems in animals [2].

However, one can expect that the process of domain shuffling could lead not only to the emergence of completely new domain combinations, but also to the independent emergence of domain combinations already present in other, even distantly related, organisms. In fact, it has been shown that certain domain combinations observed in proteins involved in innate immunity have evolved independently several times [8]. Such proteins are not descendants of an ancestor protein that already contained the domain combination(s) in question, but instead evolved independently by parallel evolution [8]. The issue of independent domain combination evolution is not only of great theoretical interest, but also important in the context of comparative functional genomics, in particular for protein function prediction. Proteins with identical architectures are oftentimes (but sometimes erroneously) viewed as orthologs with all the resulting expectations as to the similarity of their functions [18]. Yet, if the architectures are the result of two different evolutionary trajectories, they may seem like orthologs but may well not be. At the same time, the question of if and how parallel evolution of domain combinations relates to functional similarity is an important and as of yet only poorly explored one. However, here we mostly address a simpler question, namely that of how common this phenomenon is. The first study addressing this issue, based on then-available 5 eukaryotic, 11 archaeal, and 46 bacterial genomes, claimed that independent evolution of domain combinations is rare and that only 0.4% to 4.0% of proteins are the result of such evolution [19]. A more recent study based on a larger set of 28 eukaryotic, 15 archaeal, and 53 bacterial genomes suggests that between 5.6% and 12% of domain architectures (which can contain more than two domains and are thus different from domain combinations) appeared independently more than once (both between species and within species) [20]. Another study, using a large dataset of proteins, but not necessarily from complete genomes, showed a more complex picture, with such events being rare in small gene families, but more frequent in large ones [13]. Here, we return to the same question, taking advantage of a now-available, much larger set of 172 complete genomes sampling most (five out of six) eukaryotic supergroups. At the same time, we focus our analysis solely on eukaryotic genomes, mostly because the tree of life for bacteria and archaea is not well defined and because of the role lateral gene transfer is likely to play in these kingdoms.

## Results

### Domain architecture analysis in 172 eukaryotic genomes

**Figure 1:**
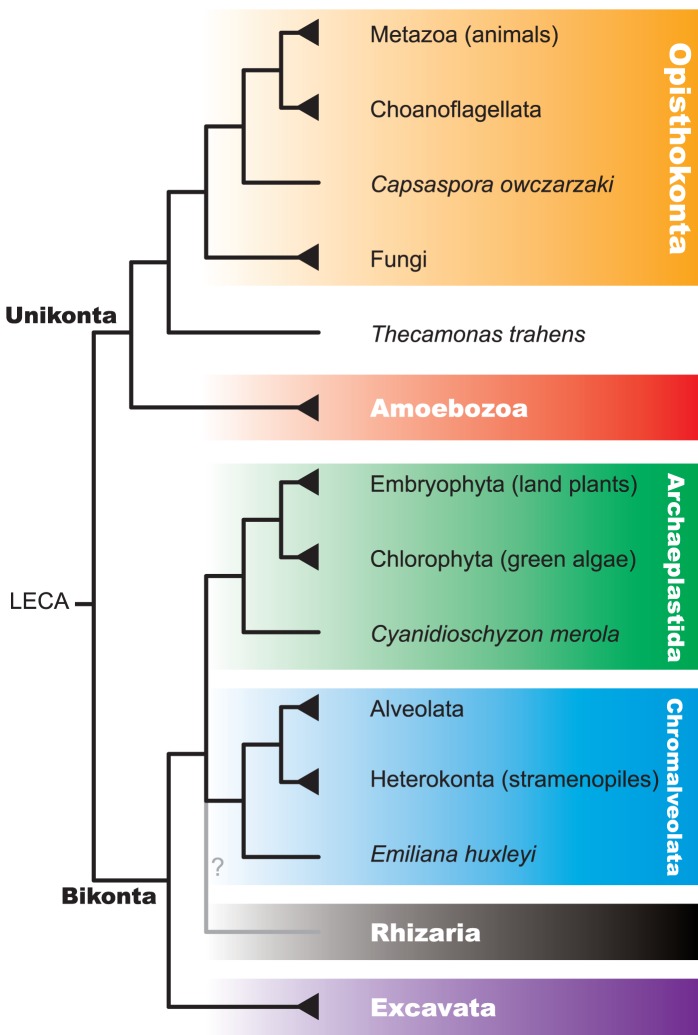
Overview of the current model of eukaryote evolution. The six “supergroups”—Opisthokonta, Amoebozoa, Archaeplastida, Chromalveolata, Rhizaria, and Excavata—are shown (the placement of Excavata is under debate) [21]–[23], [25], [29], [36], [37], [53], [54].

We have collected complete sets of predicted proteins for 172 eukaryotic genomes representing five out of six eukaryotic supergroups [21], [22]—Opisthokonta, Amoebozoa, Archaeplastida, Chromalveolata, and Excavata, with no representatives from Rhizaria [23]. In particular, we analyzed 112 genomes from Opisthokonta, namely 48 Metazoans (animals), 2 Choanoflagellata (unicellular and colonial eukaryotes, the closest living relatives of metazoa), *Capsaspora owczarzaki* as the sole representative of Filasterea (unicellular euakaryotes, forming a sister clade to Metazoa and Choanoflagellata [24]), and 61 fungi. In addition, one genome, that of *Thecamonas trahens*, represents apusozoa (flagellate protozoa, most of which feed on bacteria [25]), which are usually grouped with Opisthokonta. Three genomes in our analyses are from Amoebozoa (amoeboid protozoa), 33 from Archaeplastida (plants and relatives), 18 from Chromalveolata (a large and very diverse group of unicellular euakaryotes), and 5 from Excavata (unicellular eukaryotes, many of which lack traditional mitochondria) (see Table S1 for the full list). An overview of the current view of the phylogeny of these groups is shown in Figure 1 (a detailed phylogeny is shown in Figure S1).

All proteins from all genomes were analyzed for the presence of protein domains, as defined by the Pfam database (version 25.0) [26] and using programs from the HMMER3 package [27] with the “gathering” cutoff scores suggested by the Pfam database (see the Materials and Methods section for details). On average, 76% of all proteins have at least one domain assigned. However, the distribution of the domain coverage is very broad, with outliers at the 40% and 95% level marks (Table 1). The outliers on the low coverage level include the single-celled rodent malaria parasite *Plasmodium chabaudi* (40%), the single-celled ciliate *Tetrahymena thermophila* (44%), and the single-celled parasite *Trichomonas vaginalis* (47%). The likely explanation for these low coverages is that these genomes contain a large percentage of domains not found in the traditional model organisms, which are therefore not yet included in the Pfam database. The genome of the pufferfish *Takifugu rubripes*, on the other hand, has the highest coverage level at 94%. For the human genome, this percentage is 85%.

**Table 1:** Protein domains in 172 eukaryotic genomes.

Cutoff	Matched proteins (%) [average]	Multidomain proteins (%)	Number of domains that only appear in single-domain proteins	Number of domains that only appear in multidomain proteins	Number of domains that appear in both single- and multidomain proteins	Independent domain combination evolution (%)	Independent domain combination evolution on Pfam-clan level (%)
**“noise”**	40–94 [76]	32	1,523	576	6,095	**26.8**	**42.1**
**“gathering”**	**40–94 [76]**	**32**	**1,448**	**535**	**6,040**	**26.8**	**42.1**
**“trusted”**	40–93 [76]	32	1,457	525	5,963	**27.1**	**42.2**
**E = 10^−3^**	45–96 [81]	50	652	2,171	7,340	**30.6**	**43.7**
**E = 10^−6^**	38–94 [75]	48	694	1,316	6,337	**34.6**	**43.6**
**E = 10^−9^**	33–91 [69]	46	786	1,034	6,100	**35.9**	**43.7**

Results for the three different sets of individual bit-score cutoffs from the Pfam database [26] are shown, together with results based on three uniform E-value–based cutoffs. The results in this work are primarily based on results using the “gathering” cutoff.

Overall, 34,778 distinct domain combinations were found in the 172 genomes analyzed here. A total of 22,241 of these appear in just one genome, and only 33 (listed in Table S2) are present in all the eukaryotic genomes analyzed here and are generally involved in fundamental processes, such as transport, DNA repair, transcription, and translation. More detailed analysis is presented in the following paragraphs.

### The relationship between the number of domains and the number of domain combinations

In order to analyze the genomic domain combination content, we described each multidomain protein as a set of directed binary domain combinations. For example, a protein composed of domains A, B, and C (listed in the direction from the N-terminus to the C-terminus) is described as a set of the three binary combinations A∼B, B∼C, and A∼C. We retained information about the domain order, i.e., domain combination A∼B is not the same as B∼A. Combinations between the same domains were not included in the analysis (e.g., a protein with the architecture A-B-B would be decomposed into only one binary combination, namely A∼B). The rationale for this is that combinations between the same domains can be a result of local duplication, ancestral descent, or domain fusion and the only approach to distinguish between these would be by explicit phylogenetic analysis of each domain.

First, we simply counted how many distinct domains and domain combinations each of the 172 genomes contains. The average number of domains per protein is 1.7 for all the genomes analyzed here; however, for animal genomes this number is higher (2.0). The distribution of domains in multidomain proteins is not uniform, with 1,448 domains appearing exclusively in single-domain proteins and 535 appearing only in multidomain proteins (see Tables S3 and S4 for lists of these Pfam domains). The vast majority (6,040) of all domains appears in both single- and multidomain proteins.

**Figure 2:**
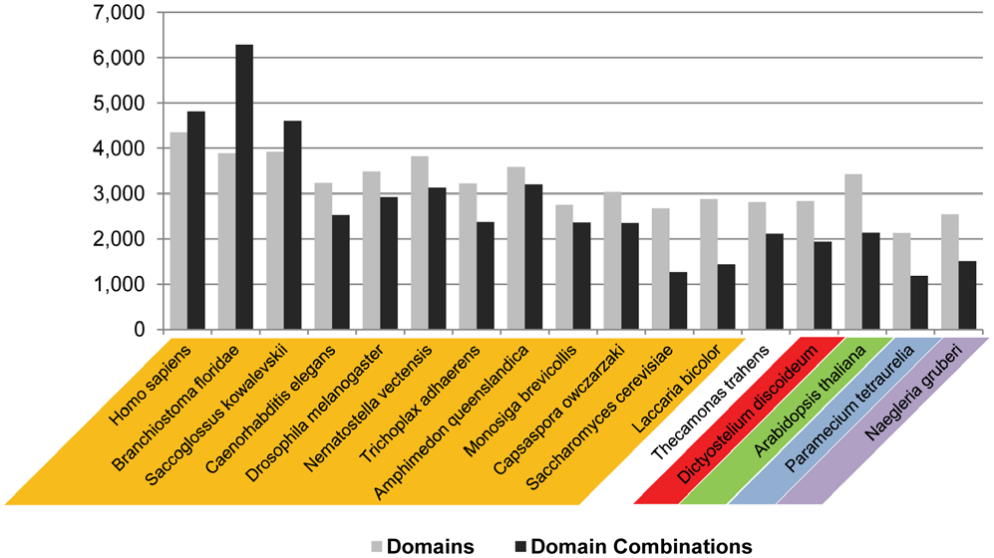
Numbers of domains and domain combinations in select species. The colors used correspond to the colors in Figure 1 (orange for Opisthokonta, red for Amoebozoa, green for Archaeplastida, blue for Chromalveolata, and purple for Excavata).

**Figure 3:**
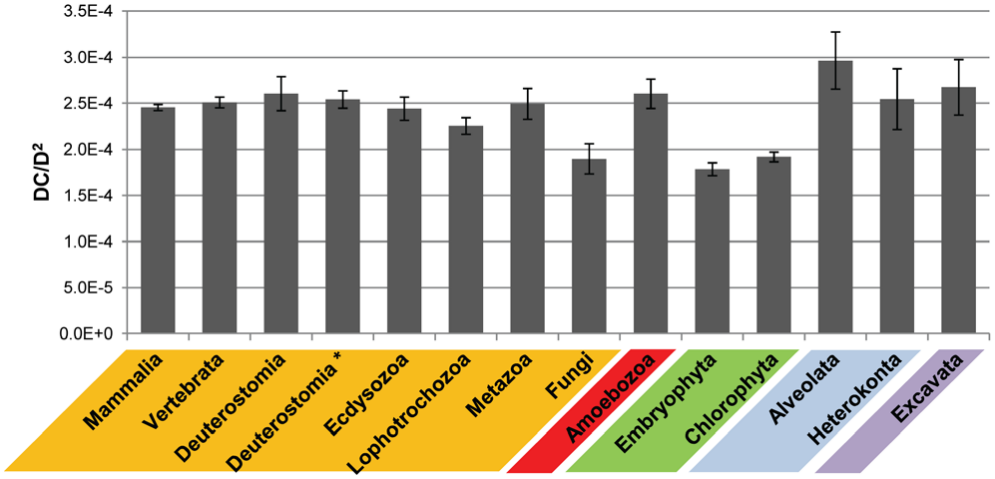
Average ratios between the numbers of domain combinations and (number of domains)^2^ for select groups of organisms. Standard deviations are shown as error bars. The asterix is used to indicate the results for Deuterostoma under exclusion of the amphioxus *Branchiostoma floridae* genome. The colors used correspond to the colors in Figure 1.

The number of distinct domains per genomes shows little variance between different organisms (with the exception of some parasitic species) and, when including inferred sets for the ancestral species, generally displays a decreasing trend in going from the last common eukaryotic ancestor to large multicellular organisms [5]. In contrast, the number of distinct domain combinations per genome varies more and shows strong correlation with the morphological complexity of their organisms, ranging from 1,178 in the free-living unicellular ciliate *Paramecium tetraurelia* to 2,372 in one of the simplest multicellular animals, *Trichoplax adhaerens* [28], to 4,821 in humans. Examples of individual domain and domain combination counts are shown in Figure 2 (data for each genome are shown in Table S5). Especially striking is the large difference in domain combination numbers between deuterostomes (which include vertebrates) and protostomes (ecdysozoa and lophotrochozoa [29]), ∼4,050 versus ∼2,650. It has been suggested that the number of domain combinations in a genome approximately grows with the number of domains squared [10]. Figure 3 shows the average ratio between the sums of the number of distinct domain combinations and the sum of (the number of distinct domains)^2^ for select groups of organisms. Even with this correction, fungi, as well as Embryophyta (land plants) and Chlorophyta (green algae), have proportionally fewer domain combinations than the other groups. On the other hand, Alveolata (a large and diverse superphylum of single-celled eukaryotes, represented in this work by genomes from ciliates and from the mostly parasitic Apicomplexa) appear to partially compensate for their limited number of domains by having a comparatively large number of domain combinations (even larger than for animals if normalized by the domain number squared).

### Clade-specific domain and domain combinations

**Figure 4:**
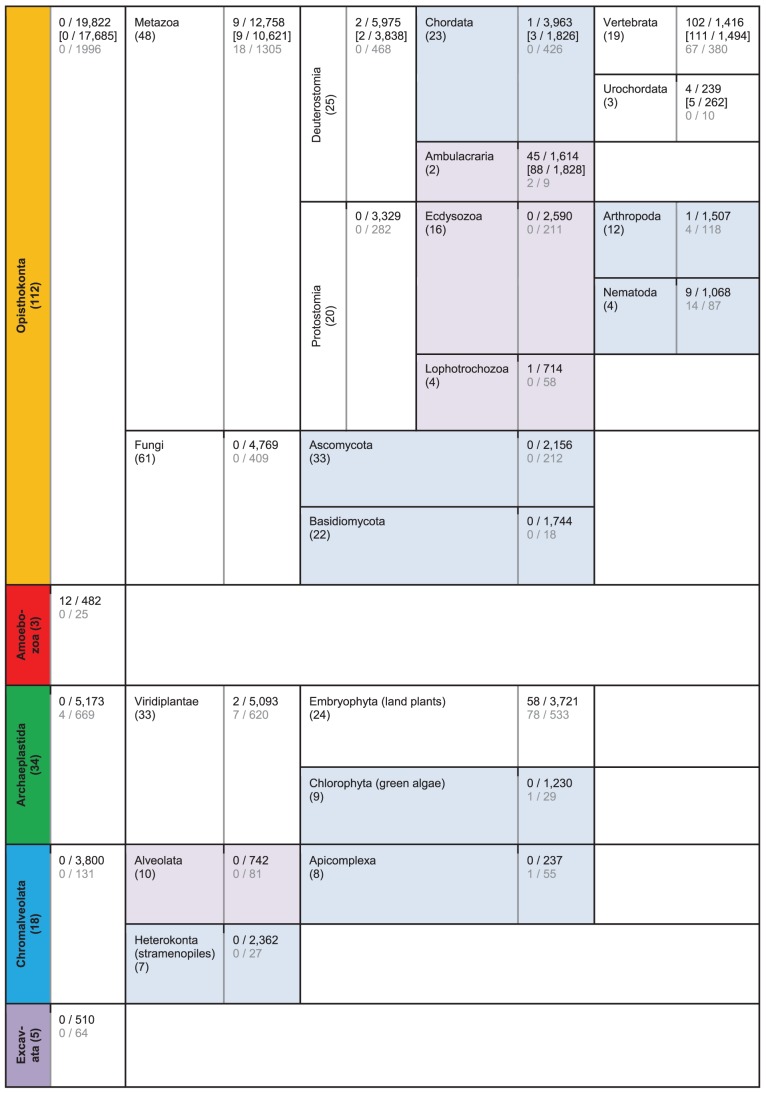
Clade-specific domains and domain combinations. This figure shows the numbers of clade-specific domain combinations (black numbers after the slash) and core domain combinations (black numbers before the slash) for select clades. Below these are the numbers of clade-specific domains (gray numbers after the slash) and core domains (gray numbers before the slash). Numbers in brackets refer to domain combination counts under exclusion of the amphioxus *Branchiostoma floridae* genome. The numbers of analyzed genomes are shown in parentheses below the clade names. For example, the 19 analyzed vertebrate genomes contain 1,416 clade-specific domain combinations, 102 of which are found in each of the 19 analyzed genomes. These 19 genomes also contain 380 clade-specific domains, out of which 67 are present in each vertebrate genome. Ambulacraria is a clade of deuterostomes that includes echinoderms and hemichordates. To facilitate comparison of different taxonomic levels, established phyla are shown with a light-blue background, whereas super-phyla have a light-purple background. This figure was made using the “gathering” cutoffs provided by Pfam. For a detailed description of parameters, see Materials and Methods. Complete counts are shown in Table S6.

As mentioned above, in our analysis of 172 genomes, 22,241 (out of 34,778) domain combinations appear only once and thus are specific to a single species. These species-specific domain combinations are relatively evenly distributed, with 95 out of the 172 analyzed genomes having between 10 and 100 domain combinations that are specific to the individual species (17 species have fewer than 10 specific domain combinations, and 60 have more than 100), with a median value of 57 (see Table S6 for complete data). Interestingly, these 95 genomes also include species for which very close relatives have been sequenced, such as human (with 41 species-specific domain combinations) and chimpanzee (9), or mouse (24) and rat (50). The chordate *Branchiostoma floridae* (amphioxus), for which no close relative has been sequenced so far, is one of the exceptions, with about 2,140 species-specific domain combinations. The next-most-prolific organisms in terms of the number of unique domain combinations are the hemichordate *Saccoglossus kowaleskii* (“acorn worm”) with about 850 species-specific domain combinations, the brown tide heterokont (stramenopile) *Aureococcus anophagefferens* (∼760), the purple sea urchin *Strongylocentrotus purpuratus* (∼720) (which, together with *S. kowaleskii*, is a member of the superphylum Ambulacraria [30]), and the sponge *Amphimedon queenslandica* (∼610). In all likelihood, in most cases the large number of unique domain combinations of these organisms is partially due to the fact that they are the sole sequenced representatives of their respective (sub-)phyla, and their domain combination counts have to be compared with those of the entire phyla, such as Arthropoda, which has about 1,500 clade-specific domain combinations (based on 12 genomes). On the kingdom level, animals clearly have the most clade-specific domain combinations (about 12,800), whereas Embryophyta (land plants) and fungi both have less than half that number. Clade-specific domain and domain combination numbers for select taxonomic groups are shown in Figure 4.

**Figure 5:**
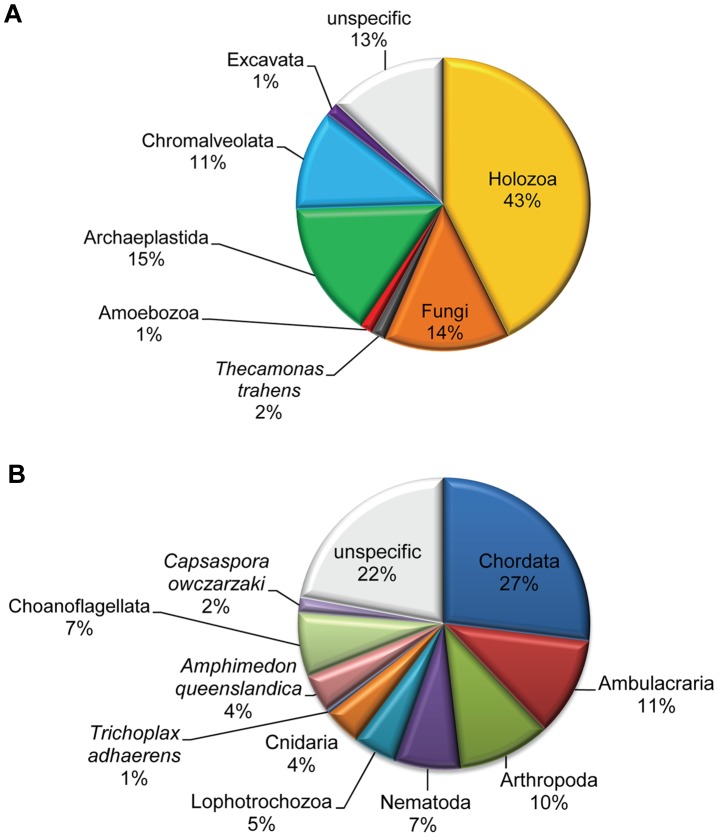
Taxonomic distribution of domain combinations. **A** shows the distribution of the 34,778 distinct domain combinations encountered in this work over the five eukaryotic “supergroups” analyzed, plus *Thecamonas trahens* (see Figure 1). **B** shows the distribution of the 14,704 Holozoa-specific domain combinations over various groups of Holozoa. See Table 2 and Table S6 for detailed numbers.

Figure 5A shows the distribution of all the 34,772 distinct domain combinations encountered in this work between the five “supergroups” covered here, plus *Thecamonas trahens* (which is related to Opisthokonta, see Figure 1). More than half (57%) of all encountered domain combinations are exclusively found in Opisthokonta, out of which 43% are specific to Holozoa (animals and their closest relatives—Choanoflagellata and *Capsaspora owczarzaki*). In contrast, only 14% are specific to fungi, despite fungi having the largest numbers of genomes analyzed in this work. Thirteen percent of all domain combinations are not supergroup-specific, i.e., they appear in at least two different supergroups. Only 2% of all domain combinations are found in representatives of all five eukaryotic supergroups analyzed in this work (listed in Table S7). Figure 5B shows the distribution of the 14,704 Holozoa-specific domain combinations over various groups of Holozoa. As expected, Chordata have the largest number of clade-specific domain combinations among Holozoa (27%). Interestingly, with 7%, the single-celled choanoflagellates are an unexpectedly large source of Holozoa-specific domain combinations, especially compared to Lophotrochozoa and Nematoda with 5% and 7%, respectively. About 20% of all Holozoa-specific domain combinations are unspecific within Holozoa at the taxonomic level used in Figure 5B (for example, appearing in both nematodes and arthropods).

We also investigated the numbers and types of domain combinations that are not only exclusive to a given clade, but also appear in *each* genome belonging to this clade. We termed such domains *core domain combinations*. Examples for selected genomes are shown in Figure 4 (see Table S6 for counts and Tables S8 and S9 for detailed lists). As expected, these numbers are generally small. For clades represented by at least 10 genomes, only vertebrates (or more precisely, Euteleostomi—“bony vertebrates”) and land plants (Embryophyta) have more than 40 core domain combinations. The majority of all clades have fewer than two core-specific domain combinations (with the obvious exceptions of extremely closely related species, such as members of the same genus, like *Arabidopsis thaliana* and *Arabidopsis lyrata*). Table 2 shows the nine metazoan-specific core domain combinations. Interestingly, these nine combinations are all involved in extracellular matrix/cell–cell adhesion (signaling) and in transcription regulation.

**Table 2:** Metazoan core domain combinations.

Pfam domains	Description
Disintegrin∼ADAM_CR	Found in **disintegrin** and metalloproteinase domain-containing proteins that may be involved in sperm-egg plasma membrane adhesion and fusion during fertilization.
Exostosin∼Glyco_transf_64	Found in Exostosin-like 1 proteins that are transmembrane glycosyltransferases of the endoplasmic reticulum and are involved in the biosynthesis of heparan sulfate proteoglycans.
FG-GAP∼Integrin_alpha2	Found in Integrin alpha-1, a receptor for laminin and collagen, and is believed to function in cell-matrix adhesion and integrin-mediated signaling pathway.
I-set∼fn3, fn3∼I-set	Found in a variety of proteins, including axon-associated and neural cell adhesion molecules (NCAMs, Fasciclins, Contactins), glycoproteins expressed on the surface of neurons, glia, skeletal muscle, and natural killer cells that have been implicated as having a role in cell–cell adhesion, neurite outgrowth, synaptic plasticity, and learning and memory.
MH1∼MH2	Found in SMAD transcription factors.
PAX∼Homeobox	Found in paired box (PAX) transcriptional regulators.
Pou∼Homeobox	Found in POU domain transcription factors.
zf-C4∼Hormone_recep	Found in a wide variety of transcription factors, including nuclear hormone receptor family members.

The 9 domain combinations exclusively found in all 48 animal genomes analyzed. For a complete list, see Table S8.

### Parallel evolution

**Figure 6:**
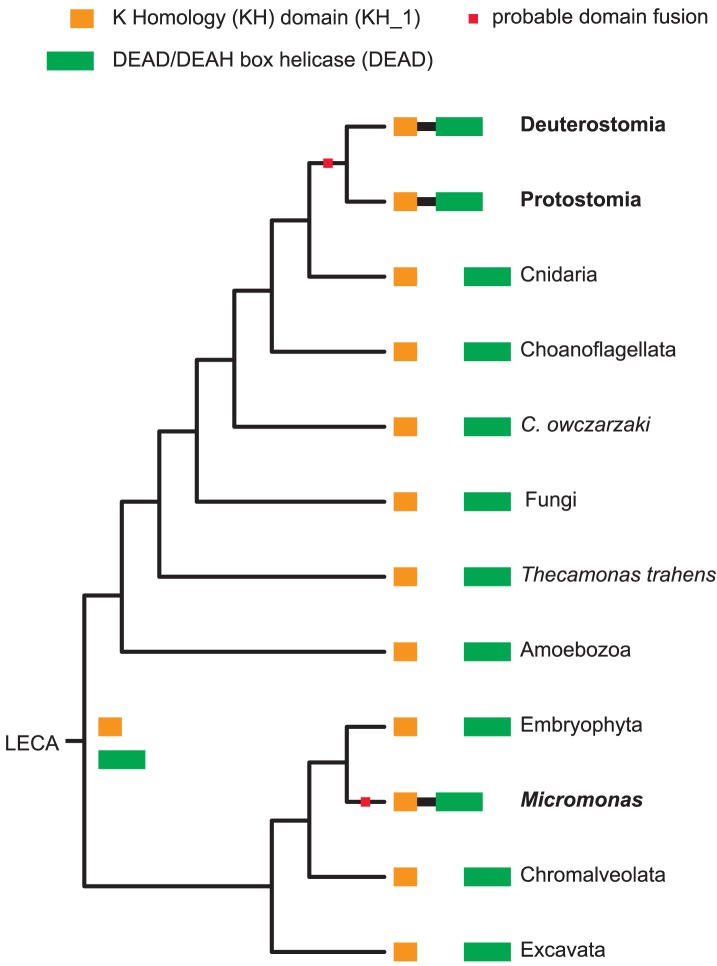
Parallel evolution of the K Homology (KH)∼DEAD/DEAH box helicase combination between Bilateria and *Micromonas* (a group of green algae). The complete diagram on which this simplified version is based is available in the supplementary materials.

Next, we investigated the evolutionary history of domain combinations—do they tend to appear once and are then inherited by the descendants (as, for example, is the case for the nine domain combinations listed in Table 2), or do they reappear independently multiple times in different branches of the tree of life? Because both fusion and fission of domains are relatively simple processes that happen as a byproduct of genome rearrangements, in sharp contrast to the appearance of protein domains themselves [5], [31], we used unweighted parsimony for the reconstruction of ancestral domain combinations [12], [32]. Unweighted parsimony assumes that domain fusion and fission are, on average, equally likely (in fact, domain fusion appears to be slightly more common than domain fission [33]–[35]). Such a parsimony analysis is based on phylogeny of all living organisms, often called the “tree of life.” Currently, the topology of “tree of life” is still disputed. Here, we use one that follows the newly emerging paradigm according to which eukaryotes can be classified into two larger clades, unikonta and bikonta [21], with details taken from literature [21]–[25], [29], [36], [37]; however, as we show later, removing controversial sections of this tree leads to quantitatively similar results. We used the Fitch algorithm in conjunction with this tree, and by minimizing the gain–loss sum [32], we calculated gains and losses for each directed domain combination. The result of this analysis, a phylogenetic tree overlaid with domain-combination gains and losses, is available as Figure S4 (Figure S2 contains data for individual domains). In order to determine whether a given directed domain combination appeared once or multiple times, we counted on how many tree nodes it (re-)appeared. For an example, see Figure 6; here, the domain combination KH (orange rectangle)∼DEAD (green rectangle) appears in bilaterian animals (deuterostomes and protostomes), as well as in a group of green algae (*Micromonas*). All the clades in-between lack this combination, even though they possess each of the individual domains. Therefore, the KH-1∼DEAD was likely to have been “rediscovered” two times (at a “cost” of two gains, as opposed to one gain and nine losses if KH-1∼DEAD were deemed ancestral).

**Figure 7:**
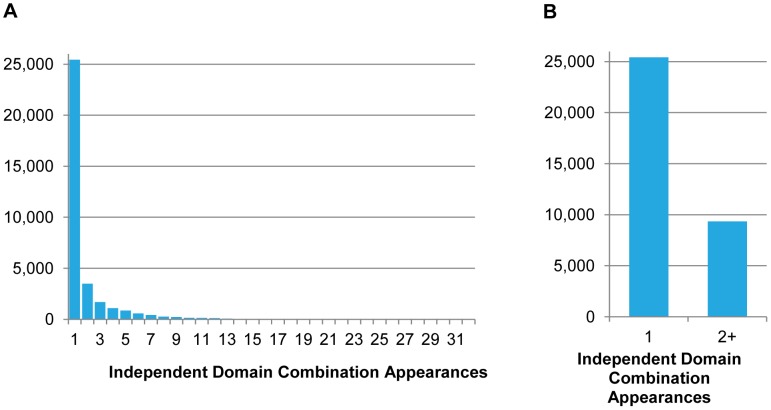
Independent domain combination evolution under an unweighted parsimony model. The histogram in **A** shows the sum for reappearing domains versus the number of reappearances. **B** is a comparison between the sum of domains that appear only once versus the sum of domains that appear more than once.

The results of analyzing each domain combination in this manner show that that a significant number of domain combinations emerged independently multiple times (Figure 7). From a total of 34,778 distinct domain combinations present in the 172 analyzed eukaryotic genomes, 25,433 were formed only once, 3,486 appeared independently twice, 1,683 three times, and 4,176 four or more times (detailed numbers are presented in Table S10; lists of domain combinations are in Table S11). The most frequently reemerging domain combinations are listed in Table 3. The total number of domain combinations that appeared independently more than once is 9,345, about 27% of the total. However, out of the total of 34,778 distinct domain combinations, 22,241 appear in only one genome (i.e., they are species-specific). Accounting for this, we can say that 75% of all recurring domain combinations have evolved independently at least once. The unexpected result of this is that for the majority of the eukaryotes analyzed here (154 out of 172), more than 50% of the domain combinations present in their genome can also be found in at least one other eukaryotic genome, not by evolutionary descent but by independent reemergence. For example, 3,431 of the 4,821 domain combinations found in the human genome (71.1%) independently evolved in at least one other species. On average, this ratio is the highest in vertebrates (70.7%) and the lowest in Excavata (43.6%). The polychaete worm *Capitella teleta* has the highest ratio (72.4%) and the obligate intracellular parasite *Encephalitozoon cuniculi* the lowest (24.4%) (detailed numbers are shown in Table S12).

**Table 3:** The most frequently reemerging domain combinations.

Number of reappearances	Domain combination	Description of domains (Pfam clans are in square brackets)	Comment
32	zf-MYND∼**SET**	MYND finger [TRASH (CL0175)]	Present in N-lysine methyltransferases.
		SET domain	
29	IMS∼IMS_HHHIMS_HHH∼IMS_C	impB/mucB/samB familyIMS family HHH motif [HHH (CL0198)]IMS family HHH motif [HHH (CL0198)]impB/mucB/samB family C-terminal	Domain architecture IMS - IMS_HHH - IMS_C is present in DNA polymerases (i.e. kappa, IV) involved in DNA repair and present in species ranging from bacteria to humans.
28	**Ank**∼zf-DHHC	ankyrin repeat [Ank (CL0465)]DHHC zinc finger domain [Zn_Beta_Ribbon (CL0167)]	Present in palmitoyltransferases.
	**Ank_2**∼RCC1	ankyrin repeat [Ank (CL0465)]Regulator of chromosome condensation (RCC1) repeat [Beta_propeller (CL0186)]	In vertebrates, the architecture Ank_2 - RCC1 - BTB is present in inhibitor of Bruton tyrosine kinase proteins.
	GST_C∼tRNA-synt_1c_C	Glutathione S-transferase (C-term.) [GST_C (CL0497)]tRNA synthetases class I (E and Q), anti-codon binding domain	Domain architecture GST_C - tRNA-synt_1c - tRNA-synt_1c_C is present in Glutamyl-tRNA synthetases.
27	GST_C∼tRNA-synt_1c	Glutathione S-transferase (C-term.) [GST_C (CL0497)]Aminoacyl tRNA synthetases, class I [tRNA_synt_I (CL0038)]	See above.
	Hexapep∼W2	Bacterial transferase hexapeptideeIF4-gamma/eIF5/eIF2-epsilon [TPR (CL0020)]	Present in translation initiation factor eIF-2B subunit epsilon.
	SMC_hinge∼SMC_N	SMC proteins Flexible Hinge DomainRecF/RecN/SMC N terminal domain [P-loop_NTPase (CL0023)]	Found in structural maintenance of chromosomes proteins, and bacterial chromosome partition proteins.
26	IBN_N∼HEAT	Importin-beta N-terminal domain [TPR (CL0020)]HEAT repeat domain (related to armadillo/beta-catenin-like repeats) [TPR (CL0020)]	Found in Importin-4, Importin subunits beta-1 and beta-4, and in Transportin (which also includes HEAT-like repeats).
	**zf-C3HC4**∼IBR	Zinc finger, C3HC4 type (RING finger) [RING (CL0229)]IBR (In Between Ring fingers) domain	Found in proteins that have been suggested to accept ubiquitin from specific E2 ubiquitin-conjugating enzymes and then transferring it to various substrates

Domains described as promiscuous in [10] are in bold.

To test whether longer proteins are more likely to contain reoccurring domain combinations than shorter ones, we compared the average lengths of proteins that contain reoccurring domain combinations to those that do not. The result is that the average length of proteins containing reappearing domain combinations is 782 residues (median: 589) and is slightly longer than that of proteins with non-reappearing domain combinations that have an average of 712 residues (median: 534). On the other hand, the average number of domains in these two groups is almost identical (∼3.3). We also compared the average lengths of domains themselves in those two groups. The results support the observation that repeated domain combinations tend to appear in longer multidomain proteins, but the preference is not very strong.

We also investigated how the specific choices of parameters affect these numbers. In particular, we tested a range of cutoff E-values (from 1e^−3^ to 1e^−15^), as well as domain-specific “trusted” and “noise” cutoff scores from the Pfam database (instead of gathering cutoff values) [26]. Under any of these conditions, the percentage of independently evolved domain combinations was at least 26% (see Table 1). Furthermore, we also assessed the effect of two alternative operational definitions of domain combinations. First, we performed our analysis under a model of undirected domain combinations (in which a domain combination is considered A∼B equivalent to B∼A). Second, we ran our analysis under a domain combination model that required domains to be adjacent to be considered a combination (e.g., a protein with A-B-C architecture contains domain combinations A∼B and B∼C, but not A∼C). Under both of these conditions, the parallel evolution percentage remained around 27% (at 27.3% and 27.9%, respectively). Since the deep topology of the eukaryotic tree of life, as well as that of some subtrees (e.g., the fungal subtree), are still topics of ongoing research (see Discussion), we also calculated the percentage of independent domain combination evolution specifically for the Metazoan and Viridiplantae (green plants) subtrees (for the reason that plant and animal evolution is comparatively well understood). If only Metazoan genomes were included in the analysis, the resulting parallel evolution percentage was 29.8%, whereas for Viridiplantae it was 19.3% (other major subtrees had percentages between these two values).

One can argue that an unexpectedly high rate of parallel evolution events is due to potential false negatives caused by highly divergent domains. In this scenario, the ancestral domain combination would be wrongly counted as having been lost and replaced by an apparently independently evolving domain combination, while in fact the “new domain” would be simply a divergent version of the old domain. To test this hypothesis, we performed the analysis analogous to one described before, but on the level of Pfam-clans (groups of domains that are believed to have originated from a common ancestor, but at much earlier point in evolution [38]). To do this, we replaced Pfam domains with their matching clan (for the roughly 47% of Pfam domains that are not members of a clan, we used the domains themselves). The resulting rate of parallel evolution of domain combinations was about 42% for all conditions tested (see Table 1). Therefore, it is unlikely that our results are simply an artifact due to highly divergent domains. The explanation of this high rate is that a proportionally large number of domain combinations that appeared only once are members of large clans.

**Figure 8:**
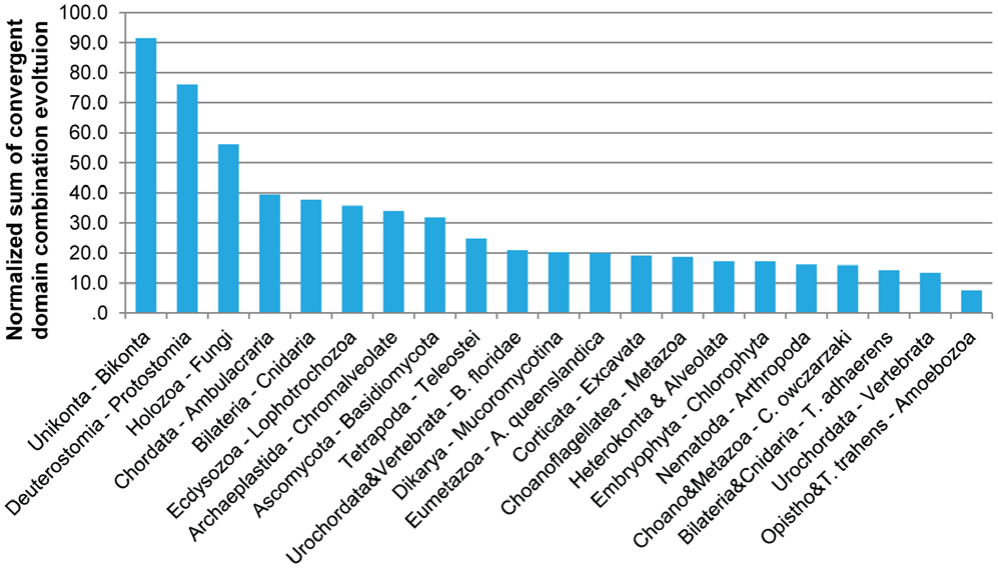
Normalized rates of independent domain combination evolution. Normalized (by the number of genomes) sums of independently evolved domain combinations across major splits on the eukaryotic tree of life are shown. “Opistho” stands for Opisthokonta and “Choano” stands for Chanoflagellatae. Ambulacraria is a clade that includes echinoderms and hemichordates.

Next, we investigated whether parallel domain combination evolution is equally prevalent in all subtrees of the eukaryotic tree of life or whether some branches differ in their propensity for parallel domain combination evolution. Related to this issue is the question of how large the evolutionary distances between pairs of independently evolved domain combinations are. For this purpose, we calculated the last common ancestor (LCA) for each pair of independently evolved domain combinations and then counted for each internal node of the eukaryotic tree of life for how many pairs of independently evolved domain combinations it represents the LCA. These counts were then normalized by the sum of species emerging from each node. The results for the nodes with the highest rates are summarized in Figure 8. The result is that the most events go back to the split between unikonts and bikonts (i.e., to the last eukaryotic common ancestor. The split between deuterostomes and protostomes has the second-highest relative rate of independent domain combination evolution. In general, we noticed that independent domain combination evolution is more prevalent in Opisthokonta, and especially in Metazoans, than in the rest of eukaryotes, even if normalized for the number of genomes.

**Figure 9:**
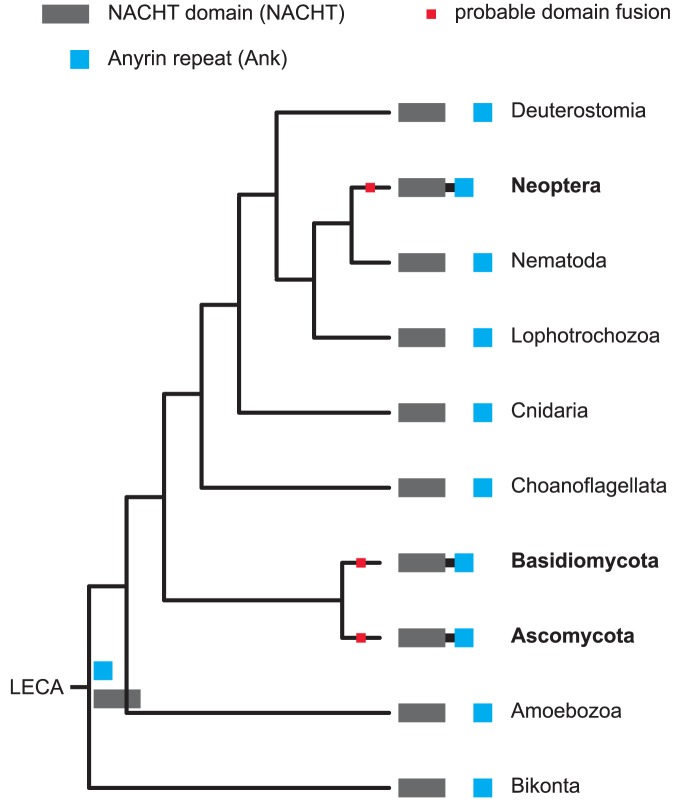
Parallel evolution of the NACHT∼Ankyrin combination between Neoptera (winged insects) and fungi. The complete diagram on which this simplified version is based is available in the supplementary materials (which explains that both major groups of fungi, Basidiomycota and Ascomycota, have one independent domain fusion event each).

**Figure 10:**
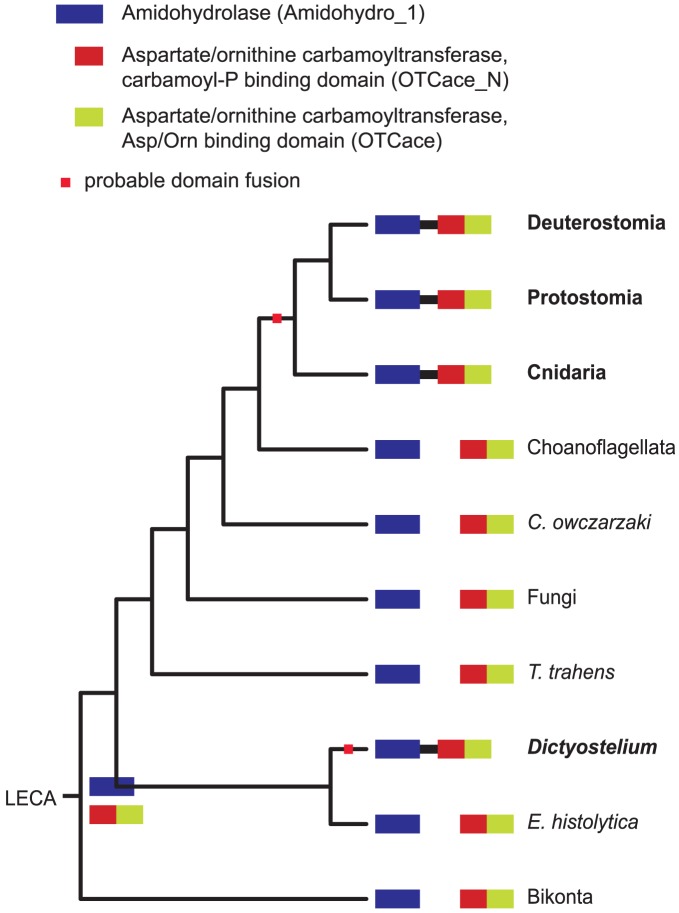
Parallel evolution of the Amidohydrolase∼Aspartate/ornithine carbamoyltransferase combination between Metazoa and *Dictyostelium*. The complete diagram on which this simplified version is based is available in the supplementary materials.

In the following, we present some examples of parallel evolution of domain combinations between animals and fungi, Amoebozoa, and green plants (see Figures 8 to 10).

An example of independent domain combination evolution between animals (Neoptera, winged insects, in this particular case) and Dikarya (that subkingdom of fungi that includes the two major phyla Ascomycota and Basidiomycota) is shown in Figure 9. The combination of a NACHT domain [39] with (a) Ankyrin repeat(s) appeared independently at least twice, once (in fact, probably more than once) in Dikarya and once in Neoptera. The nucleotide binding NACHT (present in the neuronal apoptosis inhibitory protein (NAIP), CIITA, HET-E, and TP1) domain is primarily found in proteins associated with apoptosis and innate immunity [40]. Ankyrins are a family of adaptor proteins involved in metazoan cell adhesion [41], [42]. Despite being present in numerous fungal genomes, the function and role of NACHT and Ankyrin domain proteins is unknown as of this writing. Individually, both the NACHT domain and the Ankyrin repeat are present in almost all eukaryotes (i.e., there is no particular clade in which all members lack either one of these two domains). The corresponding is true for the following two examples.

Another example of parallel evolution is the Amidohydrolase∼Aspartate/ornithine carbamoyltransferase combination that evolved independently in Metazoa and in *Dictyostelium* (Figure 10). This combination is found in the mammalian CAD protein, which is a multifunctional protein that performs multiple enzymatic activities in the *de novo* pyrimidine synthesis pathway [43].

A more-distant parallel evolution example is the evolution of the K Homology (KH)∼DEAD/DEAH box helicase combination that appeared independently in Bilateria and in Micromonas (a group of green algae) (Figure 6). The K homology (KH) domain is an evolutionarily conserved domain and is present in a wide variety of nucleic acid–binding proteins. The KH domain binds RNA and can function in RNA recognition [44]. The KH∼DEAD/DEAH box helicase combination is found in the probable ATP-dependent RNA helicase DDX43 [45], [46]. The role of the protein with this domain architecture in green algae is unknown.

### Statistical significance of the results

All the values presented here depend critically on the number and phylogenetic distribution of genomes analyzed, the size of the domain database, and the significance thresholds (and sensitivity) for domain assignments. We evaluated the effects of these on our results, especially in the light of many earlier papers reporting results of somewhat similar analyses being contradictory to our results. To understand these apparent discrepancies, we have repeated our analyses using a reduced number of genomes, different domain recognition thresholds, and smaller domain databases mimicking the approaches used in earlier works. The results from these analyses confirm that the differences between the earlier and the current results stem mostly from the increase in the number of analyzed genomes and in the size of the domain databases. For instance, using only five genomes resulted in a reemergence percentage similar to the estimates presented in ([19] and [20]). Clearly, increasing the number of the genomes in the analysis results in a large increase of this percentage. We can expect that the value reported here is a lower estimate of the real value, and with a significant growth of the number of completed eukaryotic genomes, this value could grow even further.

Similarly, we can show that other differences between our results and that of the previous analyses are mostly due to the changes in the number of genomes and the size of the domain database. For instance, analysis of five eukaryotic genomes and domain definitions from the SCOP 1.53 database [47] led to the estimate that 80% of all eukaryotic proteins are multidomain proteins [48] (similar numbers were reported in Liu), while our results suggest that this number is around 32% (Table 1). Two reasons are likely to contribute to these discrepancies. First, here we used much-more-stringent cutoff values than the unrealistically low E-value of 10^−2^ used in [48]. But even performing our analysis with an E-value cutoff of 10^−2^ instead of the domain-specific “gathering” thresholds results in a multidomain protein percentage of 52 (and a protein match range of 52% to 97%), which is still lower than reported in [33] and [49]. This effect is due to the growth of the domain databases over the last 10 years—the SCOP database has more than doubled during that time—and the specific bias in the order in which domains are added to databases such as SCOP or Pfam. For instance, central and highly promiscuous domains [10], such as kinase, PH (Pleckstrin homology), PDZ, SH3 (Src Homology 3), and AAA (ATPases Associated with diverse cellular Activities), have been studied and, as a consequence, added to the domain databases earlier than rare and less-central domains. Confirming this trend are two more-recent studies based on seven eukaryotic genomes in which the percentage of eukaryotic multidomain proteins is estimated to be 65% [49].

Unfortunately, we cannot completely exclude the effects of erroneous gene models. To partially address this problem, we performed our analysis under both inclusion as well as exclusion of the one genome with the most unusual domain combinations (that of the amphioxus *Branchiostoma floridae*). Furthermore, we performed our analysis on various subsets of all available eukaryotic genomes that are believed to be of high(er) quality (results not shown). In both cases, the effect on the resulting parallel evolution rate of 27% was negligible.

There are several simplifications made in our model that likely lead to underestimating the number of independently emerging domain combinations. First, since our analysis is not based on domain trees (evolutionary trees built for specific domains), our results do not take into account parallel domain combination evolution within a genome, i.e., between paralogs in large protein families (this is in contrast to the study performed in [20]). Second, it has previously been shown that domain fusion is more likely than domain fission [33]–[35]; thus, emergence of the same domain combinations is more likely than repeated loss of large ancestral sets of domain combinations undergoing primarily domain fission. Third, the results presented here depend to some degree on the number of genomes analyzed. Performing our analysis with a reduced number of genomes (results not shown) results in a smaller reemergence percentage (similar to the lower estimations in [19] and [20]); therefore, it is expected that with even more completed eukaryotic genomes, this percentage will grow further.

Finally, we would like to point out while the tree of life shown in Figure 1 (and in detail in Figure S1) is still disputed; this is mainly due to uncertainty regarding the placement of Rhizaria. Since our analysis does not include any genomes from this group, this controversy has no bearing on the results presented here. The second controversy concerns the placement of haptophytes (a phylum of algae), which in the model used here are considered part of Chromalveolata, but which according to recent results might form a clade with Archaeplastida [24]. In our analysis, haptophytes are represented by only one genome, *Emiliania huxleyi*, the placement of which on the tree of life has no measurable effect on the results presented here (data not shown).

## Discussion

Our analysis shows that the number of distinct domain combinations per genome varies greatly between different groups of species and increases systematically with their complexity. This increase matches the intuitive meaning of “complexity” as related to differentiation between cell types in an organism, which typically results from the interactions between multidomain regulatory processes.

The main result presented in this paper, namely the fact that at least 25% of all known and 75% of all recurring domain combinations have evolved independently, is less intuitive. On one hand, it is an obvious effect of the plasticity of eukaryotic genomes, with genome rearrangements constantly reshuffling existing domain combinations. On the other hand, it is interesting that this apparently random process leads to repeated reemergence of the same domain arrangements. Given that the genomes analyzed in this work contain a total of 8,023 distinct domains, it would allow the formation of about 64×10^6^ distinct directed domain combinations. And yet in the genomes analyzed here, we observed a total of only 34,778 domain combinations, which corresponds to only about 0.05% of the theoretical maximum. Therefore, we can speculate that the process of domain recombination is not entirely random and that organisms evolved some mechanisms that constrain the process of domain recombination in such a way that the chances of harmful, nonsensical arrangements are decreased. Here, we can only speculate about possible mechanisms to implement such constraints, but, for example, this could be achieved via the specific distribution of transposable elements and/or chromosomal locations of preferred recombination “hot spots.”

The number of times many domain combinations emerged independently is even more significant when viewed from the perspective of individual species. Over 70% of the domain combinations present in the human genome, and about 70% for all vertebrates, have evolved independently in other species at least once. This apparent discrepancy between the global and per-species averages is caused by a large number—over 22,000, unique, species-specific domain combinations, which, while rare (about 130 on average, with a median of 57) in individual species, add up to a large percentage over all species. One can argue that we are seeing two types of domain combinations: “universal, reemerging domain combinations” and “clade–specific, non-reemerging domain combinations.”

One might speculate that domains that tend to appear in independently evolved domain combinations could be functionally different from those that make up combinations that only appeared once. This seems not to be the case, though— preliminary studies using a variety of methods and tools (such as Gene Ontology term enrichment analysis) indicate that there is no significant correlation between domain function and the tendency of domains to appear in independently evolved domain combinations. Similarly, strong correlation between domain “promiscuity” [10] and presence in reemerging domain combinations could not be observed. On the other hand, the modeling of structures of several specific cases of independently emerged domain combinations indicates that surface features of individual domains could be dramatically different, suggesting dissimilar functions [8]. This interesting issue definitely requires more in-depth analysis.

Observations presented in this paper have important consequences in interpreting similarities and differences between genomes of distantly related organisms. Usually, discovery of a protein with known domain architectures in newly studied species is taken as an argument for evolutionary conservation of function of these proteins. This is of particular importance when attempting to transfer protein function from distantly related model organisms, such as from the ecdysozoans *Drosophila melanogaster* and *C. elegans,* to vertebrates, such as humans. The high rate of independent domain combination evolution between protostomes and deuterostomes (the second-largest rate; see Figure 8) is yet another reason for interpreting results from such model organisms with caution [18].

Besides estimating the rate of independent domain evolution, we also assessed the number of clade-specific domains and domain combinations. All branches of life (at all levels) have unique domain combinations (combinations not shared with other branches). Due to unequal sampling, it is difficult to compare these numbers. Nevertheless, some issues are worth mentioning. While, as expected, animals have the largest number of unique domain combinations (∼12,800, based on 48 genomes, compared to ∼4,800 in fungi based on 61 genomes and ∼3,700 in green plants based on 33 genomes), within animals there appears to be little-to-no correlation between the number of unique domain combinations and morphological complexity. For example, mammals have ∼400 unique domain combinations from 10 genomes, whereas Arthropoda have roughly three times that number (∼1,500 from 12 genomes). Clearly, the number of unique domain combinations does not explain the complexity of mammals. In this context, we introduced the concept of clade core domain combinations, combinations exclusively found in each genome of a given clade. It can be argued that such clade core domain combinations provide fundamental and distinguishing functionality for the organisms of a clade. For example, animal core domain combinations are all involved in extracellular matrix/cell–cell adhesion functions and in transcription regulation functions and are thus strongly correlated with the development of multicellular organisms.

In summary, our results stress a recurring theme—namely, that evolution is an exceedingly dynamic, and seemingly random, process. New domain combinations are being created and recreated throughout evolution. Each group of organisms (and probably even each organism) has their own solution, based on a partially shared set of building blocks (domains) to solve shared biochemical and regulatory needs.

As more and more genomes are being sequenced, we expect the percentage of independent domain combination evolution to grow even more. In fact, we expect that, with sufficient data available, the following paradigm of evolution at the domain level will emerge. Major clades (such as animals) have a relatively small set of distinguishing core domain combinations that are essential and defining for members of that clade (such as developmental programs and cell–cell adhesion for animals). Outside of these hierarchical sets of core domain combinations (such as for eukaryotes, animals, and vertebrates), all domains are randomly undergoing reshuffling, and the vast majority keep reemerging and disappearing both over species space and over time, with the exception of various small sets of core domain combinations.

## Materials and Methods

Protein predictions for 172 completed eukaryotic genomes were downloaded from a variety of sources (for details, see Table S1) and analyzed for domain content against the hidden Markov models (HMMs) from the Pfam domain database (version 25.0) using hmmpfam from the HMMER software package (version 3.0) [26], [27]. For score thresholds, we primarily used the per-domain “gathering” cutoff bit scores (“GA2”) from the Pfam database (these cutoffs are used by Pfam to determine which sequences get included in Pfam full alignments). Domains associated with viruses, transposons, and bacteriophages were ignored. For overlapping domains, only the domain with the lowest E-value was retained. The domains of multidomain proteins were decomposed into all possible pairs of directed binary combinations; combinations between identical domains were ignored. Based on these preprocessing steps, lists of domains and domain combinations were created for each genome analyzed and then mapped onto corresponding external nodes of the eukaryotic evolutionary tree (see Figure S1). The presence and absence of domains and domain combinations for each internal tree node were inferred under unweighted parsimony using the Fitch algorithm for domain combinations [32], [50] and using the Dollo parsimony for individual domains [5], [51].This allowed us to count how many times each domain combination appeared. In order to test the robustness of the results, we performed the analyses with various parameters. For example, we tested filtering the predicted domains by E-values ranging from 10^−3^ to 10^−15^ and/or filtering using the lists of domain-specific score cutoff values used by the Pfam database (“trusted,” “gathering,” and “noise” cutoffs, with trusted cutoffs being the most stringent). Furthermore, we tested the effects of ignoring overlapping domains. We were unable to find a combination of these settings that would significantly change the numbers presented here and invalidate our conclusions. The preprocessing steps, the unweighted Fitch parsimony, the Dollo parsimony, and basic ancestral GO term analyses were performed by software of our own design [52].

## Supporting Information

Figure S1 **The phylogenetic tree used.** This shows the detailed tree that was used in the parsimony analysis and on which Figure 1 is based.
(PDF)

Figure S2 **Domain gains and losses during eukaryote evolution.** phyloXML [55] formatted file, viewable with Archaeopteryx software [56]. Domain gains and losses are inferred under Dollo parsimony [5]. Summary of conditions used: protein predictions as listed in Table S1, domain models from Pfam 25.0, analyzed with HMMER 3.0, Pfam “gathering” cutoffs.
(BZ2)

Figure S3 **Domain combination gains and losses during eukaryote evolution.** phyloXML [55] formatted file, viewable with Archaeopteryx software [56]. Domain combination gains and losses are inferred under unweighted parsimony [32], Summary of conditions used: protein predictions as listed in Table S1, domain models from Pfam 25.0, analyzed with HMMER 3.0, Pfam “gathering” cutoffs.
(BZ2)

Figure S4 **Parallel evolution of the NACHT∼Ankyrin combination between Neoptera and fungi.** Tree nodes in which the domain combination in question has been inferred to be present are highlighted in green. Summary of conditions used: protein predictions as listed in Table S1, domain models from Pfam 25.0, analyzed with HMMER 3.0, Pfam “gathering” cutoffs.
(PDF)

Figure S5 **Parallel evolution of the Amidohydrolase∼Aspartate/ornithine carbamoyltransferase combination between Metazoa and** ***Dictyostelium*** **.** Tree nodes in which the domain combination in question has been inferred to be present are highlighted in green. Summary of conditions used: protein predictions as listed in Table S1, domain models from Pfam 25.0, analyzed with HMMER 3.0, Pfam “gathering” cutoffs.
(PDF)

Figure S6 **Parallel evolution of the K Homology (KH)∼DEAD/DEAH box helicase combination between Bilateria and** ***Micromonas*** **.** Tree nodes in which the domain combination in question has been inferred to be present are highlighted in green. Summary of conditions used: protein predictions as listed in Table S1, domain models from Pfam 25.0, analyzed with HMMER 3.0, Pfam “gathering” cutoffs.
(PDF)

Table S1 **Genomes analyzed.**
(XLS)

Table S2 **The 33 domain combinations present in all 172 eukaryotic genomes analyzed.** This table shows the 33 domain combinations that are present in all 172 eukaryotic genomes analyzed in this work together with typical proteins in which these domain combinations appear. Summary of conditions used: protein predictions as listed in Table S1, domain models from Pfam 25.0, analyzed with HMMER 3.0, Pfam “gathering” cutoffs.
(XLS)

Table S3 **Domains appearing only in single-domain proteins.** This table shows the 1,448 domains that appear only in single-domain proteins in the 172 genomes analyzed. Summary of conditions used: protein predictions as listed in Table S1, domain models from Pfam 25.0, analyzed with HMMER 3.0, Pfam “gathering” cutoffs.
(XLS)

Table S4 **Domains appearing only in multidomain proteins.** This table shows the 535 domains that appear only in multidomain proteins in the 172 genomes analyzed. Summary of conditions used: protein predictions as listed in Table S1, domain models from Pfam 25.0, analyzed with HMMER 3.0, Pfam “gathering” cutoffs.
(XLS)

Table S5 **Domain and domain combination counts in extant species.** This table shows the numbers of distinct domains and domain combinations for the 172 genomes analyzed (color coded according to taxonomic groups and inversely sorted according to the number of domain combinations). The ratio (domain combinations)/(domains^2^) is also shown, Summary of conditions used: protein predictions as listed in Table S1, domain models from Pfam 25.0, analyzed with HMMER 3.0, Pfam “gathering” cutoffs.
(XLS)

Table S6 **Core and clade-specific domain and domain combination numbers.** This shows the numbers of core, as well as clade- and species-specific, domains and domain combinations. Summary of conditions used: protein predictions as listed in Table S1, domain models from Pfam 25.0, analyzed with HMMER 3.0, Pfam “gathering” cutoffs. The phylogenetic tree used is shown in Figure S1 and simplified in Figure 1.
(XLS)

Table S7 **Domain combinations appearing in representatives of each of the five eukaryotic supergroups analyzed.** Each domain combination listed in this table appears in at least one member of each of the five eukaryotic supergroups analyzed in this work. Summary of conditions used: protein predictions as listed in Table S1, domain models from Pfam 25.0, analyzed with HMMER 3.0, Pfam “gathering” cutoffs. The phylogenetic tree used is shown in Figure S1 and simplified in Figure 1.
(XLS)

Table S8 **List of core domain combinations.** Summary of conditions used: protein predictions as listed in Table S1, domain models from Pfam 25.0, analyzed with HMMER 3.0, Pfam “gathering” cutoffs. The phylogenetic tree used is shown in Figure S1 and simplified in Figure 1.
(XLS)

Table S9 **List of core domains.** Summary of conditions used: protein predictions as listed in Table S1, domain models from Pfam 25.0, analyzed with HMMER 3.0, Pfam “gathering” cutoffs. The phylogenetic tree used is shown in Figure S1 and simplified in Figure 1.
(XLS)

Table S10 **Numbers of reappearing domain combinations.** Summary of conditions used: protein predictions as listed in Table S1, domain models from Pfam 25.0, analyzed with HMMER 3.0, Pfam “gathering” cutoffs. The phylogenetic tree used is shown in Figure S1 and simplified in Figure 1.
(XLS)

Table S11 **List of reappearing domain combinations.** Summary of conditions used: protein predictions as listed in Table S1, domain models from Pfam 25.0, analyzed with HMMER 3.0, Pfam “gathering” cutoffs. The phylogenetic tree used is shown in Figure S1 and simplified in Figure 1.
(XLS)

Table S12 **Numbers of domain combinations per genome which independently evolved in a different genome at least once.** Summary of conditions used: protein predictions as listed in Table S1, domain models from Pfam 25.0, analyzed with HMMER 3.0, Pfam “gathering” cutoffs. The phylogenetic tree used is shown in Figure S1 and simplified in Figure 1.
(XLS)

## References

[1] Moore AD, Björklund ÅK, Ekman D, Bornberg-Bauer E, Elofsson A (2008) Arrangements in the modular evolution of proteins. Trends Biochem Sci 33: 444–451. 18656364 10.1016/j.tibs.2008.05.008

[2] Itoh M, Nacher JC, Kuma K-i, Goto S, Kanehisa M (2007) Evolutionary history and functional implications of protein domains and their combinations in eukaryotes. Genome Biol 8: R121. 17588271 10.1186/gb-2007-8-6-r121 PMC2394772

[3] Peisajovich SG, Garbarino JE, Wei P, Lim WA (2010) Rapid diversification of cell signaling phenotypes by modular domain recombination. Science 328: 368–372. 20395511 10.1126/science.1182376 PMC2975375

[4] Jin J, Xie X, Chen C, Park JG, Stark C, et al (2009) Eukaryotic protein domains as functional units of cellular evolution. Sci Signal 2: ra76. 19934434 10.1126/scisignal.2000546

[5] Zmasek CM, Godzik A (2011) Strong functional patterns in the evolution of eukaryotic genomes revealed by the reconstruction of ancestral protein domain repertoires. Genome Biol 12: R4. 21241503 10.1186/gb-2011-12-1-r4 PMC3091302

[6] Koonin EV, Wolf YI, Karev GP (2002) The structure of the protein universe and genome evolution. Nature 420: 218–223. 12432406 10.1038/nature01256

[7] Tordai H, Nagy A, Farkas K, Bányai L, Patthy L (2005) Modules, multidomain proteins and organismic complexity. The FEBS Journal 272: 5064–5078. 16176277 10.1111/j.1742-4658.2005.04917.x

[8] Zhang Q, Zmasek CM, Godzik A (2010) Domain architecture evolution of pattern-recognition receptors. Immunogenetics 62: 263–272. 20195594 10.1007/s00251-010-0428-1 PMC2858798

[9] Marcotte EM (1999) Detecting Protein Function and Protein-Protein Interactions from Genome Sequences. Science 285: 751–753. 10427000 10.1126/science.285.5428.751

[10] Basu MK, Carmel L, Rogozin IB, Koonin EV (2008) Evolution of protein domain promiscuity in eukaryotes. Genome Res 18: 449–461. 18230802 10.1101/gr.6943508 PMC2259109

[11] Weiner J, Moore AD, Bornberg-Bauer E (2008) Just how versatile are domains? BMC Evol Biol 8: 285. 18854028 10.1186/1471-2148-8-285 PMC2588589

[12] Fong JH, Geer LY, Panchenko AR, Bryant SH (2007) Modeling the evolution of protein domain architectures using maximum parsimony. J Mol Biol 366: 307–315. 17166515 10.1016/j.jmb.2006.11.017 PMC1858635

[13] Przytycka T, Davis G, Song N, Durand D (2006) Graph theoretical insights into evolution of multidomain proteins. J Comput Biol 13: 351–363. 16597245 10.1089/cmb.2006.13.351 PMC1815482

[14] Wang M, Caetano-Anollés G (2009) The evolutionary mechanics of domain organization in proteomes and the rise of modularity in the protein world. Structure 17: 66–78. 19141283 10.1016/j.str.2008.11.008

[15] Chothia C, Gough J, Vogel C, Teichmann Sa (2003) Evolution of the protein repertoire. Science 300: 1701–1703. 12805536 10.1126/science.1085371

[16] Patthy L (2003) Modular assembly of genes and the evolution of new functions. Genetica 118: 217–231. 12868611

[17] Kawashima T, Kawashima S, Tanaka C, Murai M, Yoneda M, et al (2009) Domain shuffling and the evolution of vertebrates. Genome Res 19: 1393–1403. 19443856 10.1101/gr.087072.108 PMC2720177

[18] Zmasek CM, Eddy SR (2002) RIO: analyzing proteomes by automated phylogenomics using resampled inference of orthologs. BMC Bioinformatics 3: 14. 12028595 10.1186/1471-2105-3-14 PMC116988

[19] Gough J (2005) Convergent evolution of domain architectures (is rare). Bioinformatics 21: 1464–1471. 15585523 10.1093/bioinformatics/bti204

[20] Forslund K, Henricson A, Hollich V, Sonnhammer ELL (2008) Domain Tree-Based Analysis of Protein Architecture Evolution. Mol Biol Evol 25: 254–264. 18025066 10.1093/molbev/msm254

[21] Hampl V, Hug L, Leigh JW, Dacks JB, Lang BF, et al (2009) Phylogenomic analyses support the monophyly of Excavata and resolve relationships among eukaryotic “supergroups”. Proc Natl Acad Sci U S A 106: 3859–3864. 19237557 10.1073/pnas.0807880106 PMC2656170

[22] Parfrey LW, Barbero E, Lasser E, Dunthorn M, Bhattacharya D, et al (2006) Evaluating Support for the Current Classification of Eukaryotic Diversity. PLoS Genet 2: e220. 17194223 10.1371/journal.pgen.0020220 PMC1713255

[23] Burki F, Pawlowski J (2006) Monophyly of Rhizaria and Multigene Phylogeny of Unicellular Bikonts. Mol Biol Evol 23: 1922–1930. 16829542 10.1093/molbev/msl055

[24] Burki F, Shalchian-Tabrizi K, Pawlowski J (2008) Phylogenomics reveals a new ‘megagroup’ including most photosynthetic eukaryotes. Biol Lett 4: 366–369. 18522922 10.1098/rsbl.2008.0224 PMC2610160

[25] Cavalier-Smith T, Chao EE (2010) Phylogeny and evolution of apusomonadida (protozoa: apusozoa): new genera and species. Protist 161: 549–576. 20537943 10.1016/j.protis.2010.04.002

[26] Finn RD, Mistry J, Tate J, Coggill P, Heger A, et al (2010) The Pfam protein families database. Nucleic Acids Res 38: D211–222. 19920124 10.1093/nar/gkp985 PMC2808889

[27] Eddy SR (2009) A new generation of homology search tools based on probabilistic inference. In: Genome informatics International Conference on Genome Informatics 23: 205–211. 20180275

[28] Srivastava M, Begovic E, Chapman J, Putnam NH, Hellsten U, et al (2008) The Trichoplax genome and the nature of placozoans. Nature 454: 955–960. 18719581 10.1038/nature07191

[29] Halanych KM (2004) The New View of Animal Phylogeny. Annual Review of Ecology, Evolution, and Systematics 35: 229–256.

[30] Furlong RF, Holland PWH (2002) Bayesian phylogenetic analysis supports monophyly of ambulacraria and of cyclostomes. Zoological science 19: 593–599. 12130812 10.2108/zsj.19.593

[31] Pasek S, Risler J-L, Brézellec P (2006) Gene fusion/fission is a major contributor to evolution of multi-domain bacterial proteins. Bioinformatics 22: 1418–1423. 16601004 10.1093/bioinformatics/btl135

[32] Felsenstein J (2004) Inferring Phylogenies. Sunderland, MA: Sinauer Associates.

[33] Björklund AK, Ekman D, Light S, Frey-Skött J, Elofsson A (2005) Domain rearrangements in protein evolution. J Mol Biol 353: 911–923. 16198373 10.1016/j.jmb.2005.08.067

[34] Kummerfeld SK, Teichmann SA (2005) Relative rates of gene fusion and fission in multi-domain proteins. Trends Genet 21: 25–30. 15680510 10.1016/j.tig.2004.11.007

[35] Nagy A, Patthy L (2011) Reassessing Domain Architecture Evolution of Metazoan Proteins: The Contribution of Different Evolutionary Mechanisms. Genes 2: 578–598. 24710211 10.3390/genes2030578 PMC3927616

[36] James TY, Kauff F, Schoch CL, Matheny PB, Hofstetter V, et al (2006) Reconstructing the early evolution of Fungi using a six-gene phylogeny. Nature 443: 818–822. 17051209 10.1038/nature05110

[37] Hibbett DS, Binder M (2002) Evolution of complex fruiting-body morphologies in homobasidiomycetes. Proceedings Biological sciences/The Royal Society 269: 1963–1969. 10.1098/rspb.2002.2123 PMC1691125 12396494

[38] Finn RD, Mistry J, Schuster-Bockler B, Griffiths-Jones S, Hollich V, et al (2006) Pfam: clans, web tools and services. Nucleic Acids Res 34: D247–251. 16381856 10.1093/nar/gkj149 PMC1347511

[39] Koonin EV, Aravind L (2000) The NACHT family - a new group of predicted NTPases implicated in apoptosis and MHC transcription activation. Trends Biochem Sci 25: 223–224. 10782090 10.1016/s0968-0004(00)01577-2

[40] Martinon F, Tschopp J (2004) Inflammatory Caspases: Linking an Intracellular Innate Immune System to Autoinflammatory Diseases. Cell 117: 561–574. 15163405 10.1016/j.cell.2004.05.004

[41] Bennett V, Baines aJ (2001) Spectrin and ankyrin-based pathways: metazoan inventions for integrating cells into tissues. Physiol Rev 81: 1353–1392. 11427698 10.1152/physrev.2001.81.3.1353

[42] Mosavi LK, Cammett TJ, Desrosiers DC, Peng Z-Y (2004) The ankyrin repeat as molecular architecture for protein recognition. Protein Sci 13: 1435–1448. 15152081 10.1110/ps.03554604 PMC2279977

[43] Iwahana H, Fujimura M, Ii S, Kondo M, Moritani M, et al (1996) Molecular cloning of a human cDNA encoding a trifunctional enzyme of carbamoyl-phosphate synthetase-aspartate transcarbamoylase-dihydroorotase in de Novo pyrimidine synthesis. Biochem Biophys Res Commun 219: 249–255. 8619816 10.1006/bbrc.1996.0213

[44] García-Mayoral MF, Hollingworth D, Masino L, Díaz-Moreno I, Kelly G, et al (2007) The structure of the C-terminal KH domains of KSRP reveals a noncanonical motif important for mRNA degradation. Structure 15: 485–498. 17437720 10.1016/j.str.2007.03.006

[45] Martelange V, Smet CD, Plaen ED, Lurquin C, Boon T (2000) Identification on a Human Sarcoma of Two New Genes with Tumor-specific Expression. Cancer Res 60: 3848–3855. 10919659

[46] Parsyan A, Svitkin Y, Shahbazian D, Gkogkas C, Lasko P, et al (2011) mRNA helicases: the tacticians of translational control. Nature Reviews Molecular Cell biology 12: 235–245. 21427765 10.1038/nrm3083

[47] Andreeva A, Howorth D, Chandonia J-M, Brenner SE, Hubbard TJP, et al (2008) Data growth and its impact on the SCOP database: new developments. Nucleic Acids Res 36: D419–425. 18000004 10.1093/nar/gkm993 PMC2238974

[48] Apic G, Gough J, Teichmann SA (2001) Domain combinations in archaeal, eubacterial and eukaryotic proteomes1. J Mol Biol 310: 311–325. 11428892 10.1006/jmbi.2001.4776

[49] Liu J, Rost B (2004) CHOP proteins into structural domain-like fragments. Proteins 55: 678–688. 15103630 10.1002/prot.20095

[50] Fitch WM (1971) Towards defining the course of evolution: minimum change for a specific tree topology. Syst Zool 20: 406–416.

[51] Farris JS (1977) Phylogenetic Analysis Under Dollo's Law. Syst Zool 26: 77–88.

[52] Zmasek CM (2012) Surfacing, a tool for the functional analysis of domainome/genome evolution, version 2.002. Available: http://www.phylosoft.org/forester/applications/surfacing/. Accessed 22 Jan 2012.

[53] Shalchian-Tabrizi K, Minge Ma, Espelund M, Orr R, Ruden T, et al (2008) Multigene phylogeny of choanozoa and the origin of animals. PLoS ONE 3: e2098. 18461162 10.1371/journal.pone.0002098 PMC2346548

[54] Roger AJ, Simpson AGB (2009) Evolution: revisiting the root of the eukaryote tree. Curr Biol 19: R165–167. 19243692 10.1016/j.cub.2008.12.032

[55] Han MV, Zmasek CM (2009) phyloXML: XML for evolutionary biology and comparative genomics. BMC Bioinformatics 10: 356. 19860910 10.1186/1471-2105-10-356 PMC2774328

[56] Zmasek CM (2012) Archaeopteryx: Visualization, Analysis, and Editing of Phylogenetic Trees, version 0.972. Available: http://www.phylosoft.org/archaeopteryx/. Accessed 22 Jan 2012.

