# Supplementary figures and images for: This Déjà Vu Feeling—Analysis of Multidomain Protein Evolution in Eukaryotic Genomes
Source: PLoS Comput Biol. 2012 Nov 15;8(11):e1002701. doi: 10.1371/journal.pcbi.1002701 (PMC3499355; doi:10.1371/journal.pcbi.1002701)

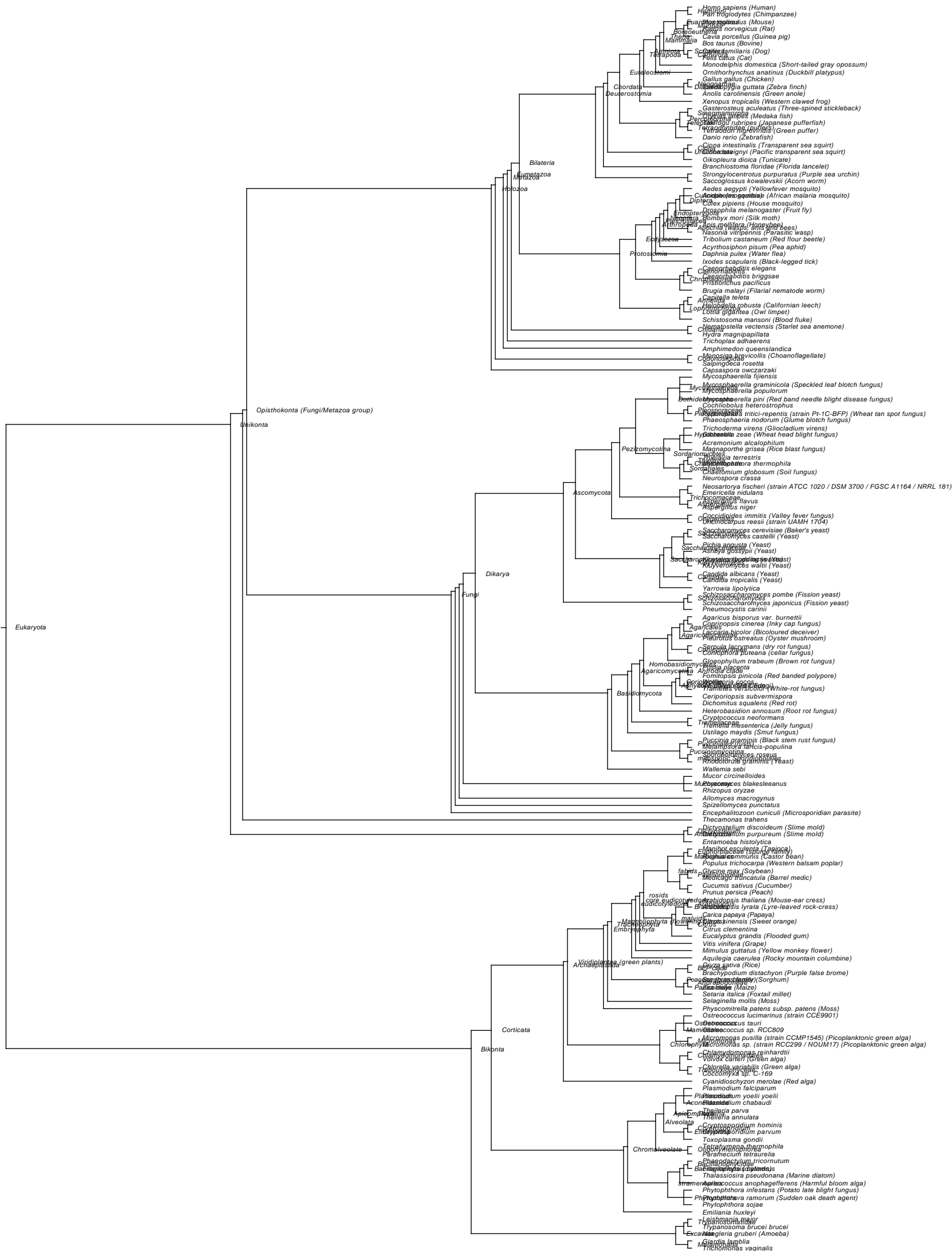

Supplement: Figure S1 — The phylogenetic tree used. This shows the detailed tree that was used in the parsimony analysis and on which Figure 1 is based. (PDF) [file pcbi.1002701.s001.pdf]

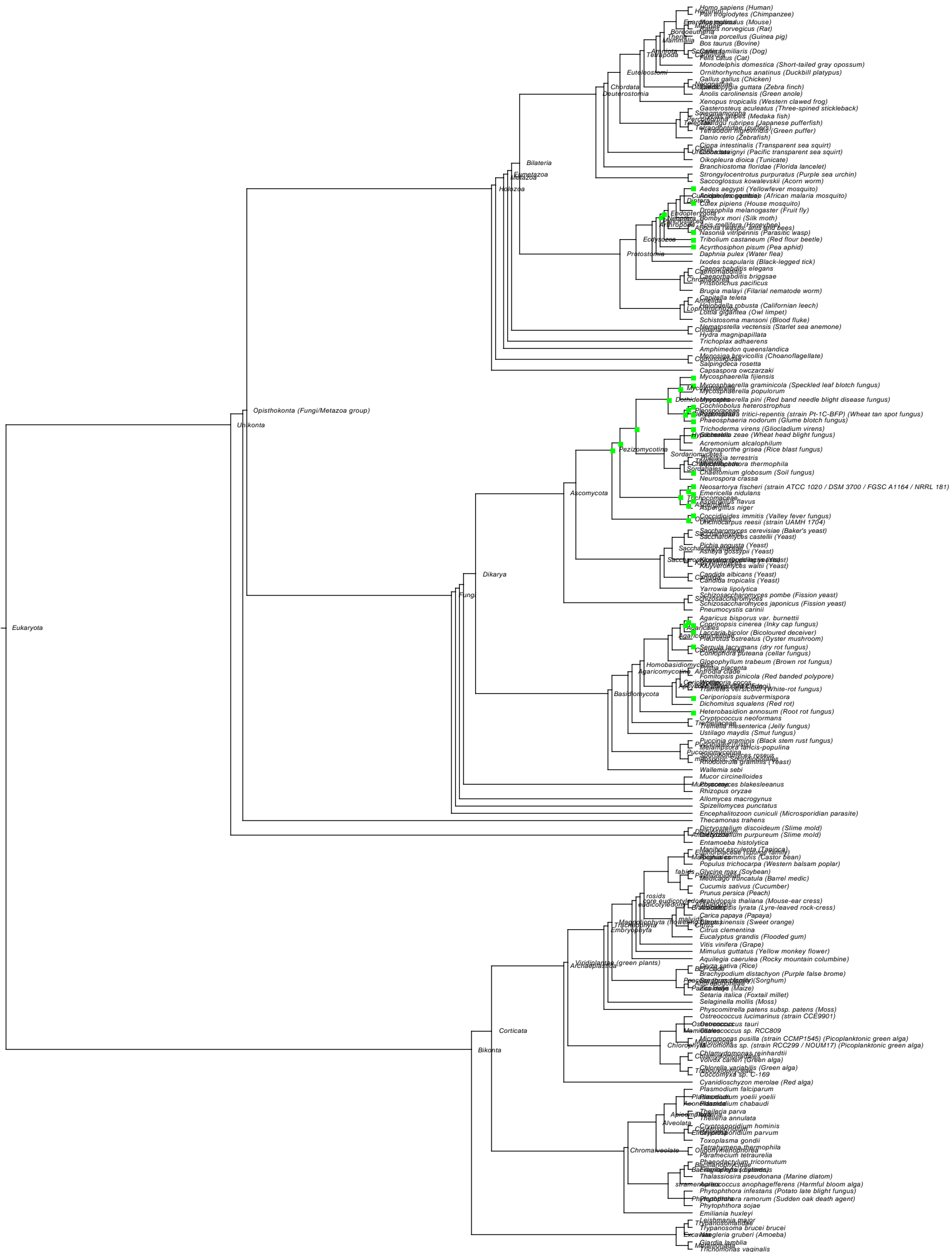

Supplement: Figure S4 — Parallel evolution of the NACHT∼Ankyrin combination between Neoptera and fungi. Tree nodes in which the domain combination in question has been inferred to be present are highlighted in green. Summary of conditions used: protein predictions as listed in Table S1, domain models from Pfam 25.0, analyzed with HMMER 3.0, Pfam “gathering” cutoffs. (PDF) [file pcbi.1002701.s004.pdf]

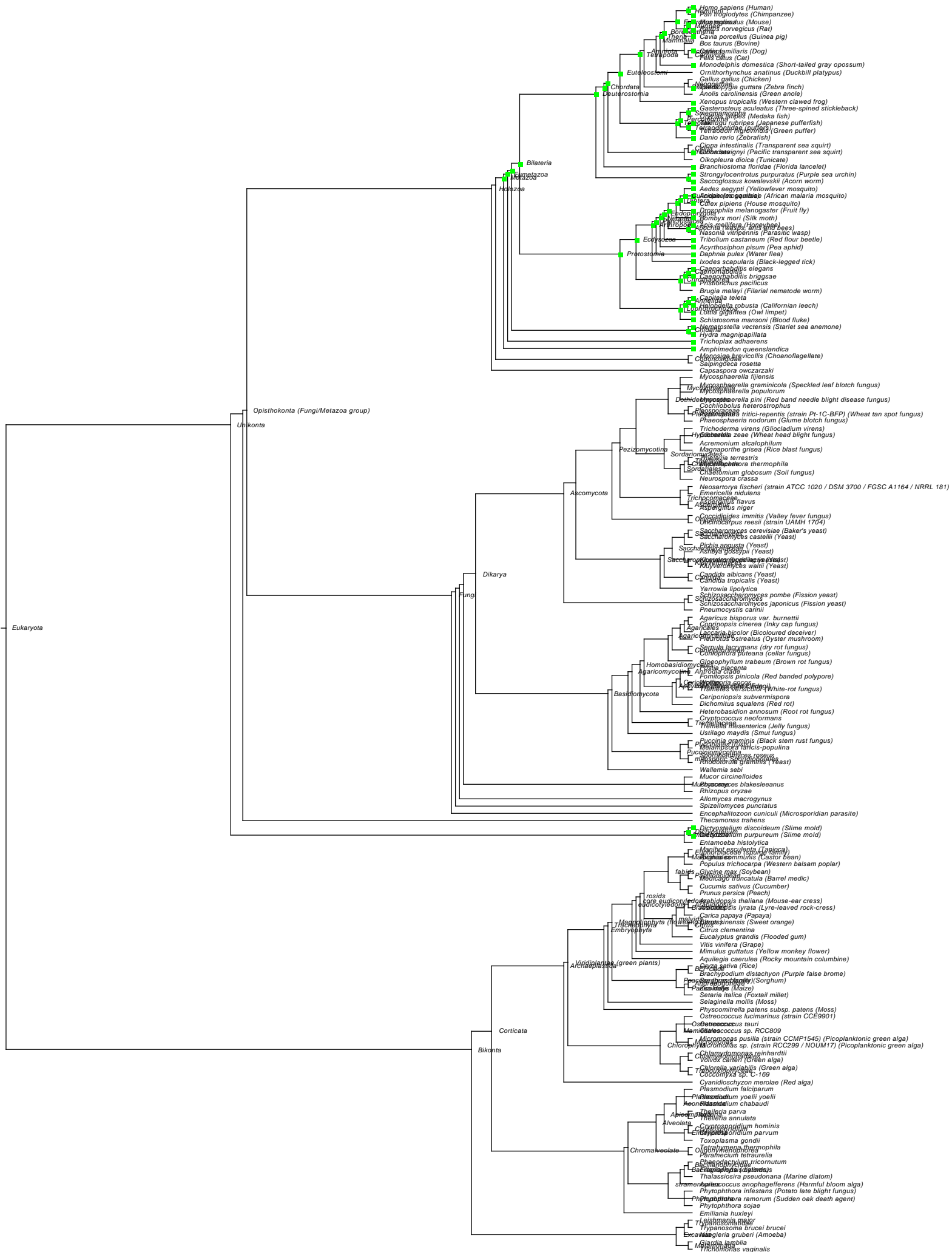

Supplement: Figure S5 — Parallel evolution of the Amidohydrolase∼Aspartate/ornithine carbamoyltransferase combination between Metazoa and Dictyostelium . Tree nodes in which the domain combination in question has been inferred to be present are highlighted in green. Summary of conditions used: protein predictions as listed in Table S1, domain models from Pfam 25.0, analyzed with HMMER 3.0, Pfam “gathering” cutoffs. (PDF) [file pcbi.1002701.s005.pdf]

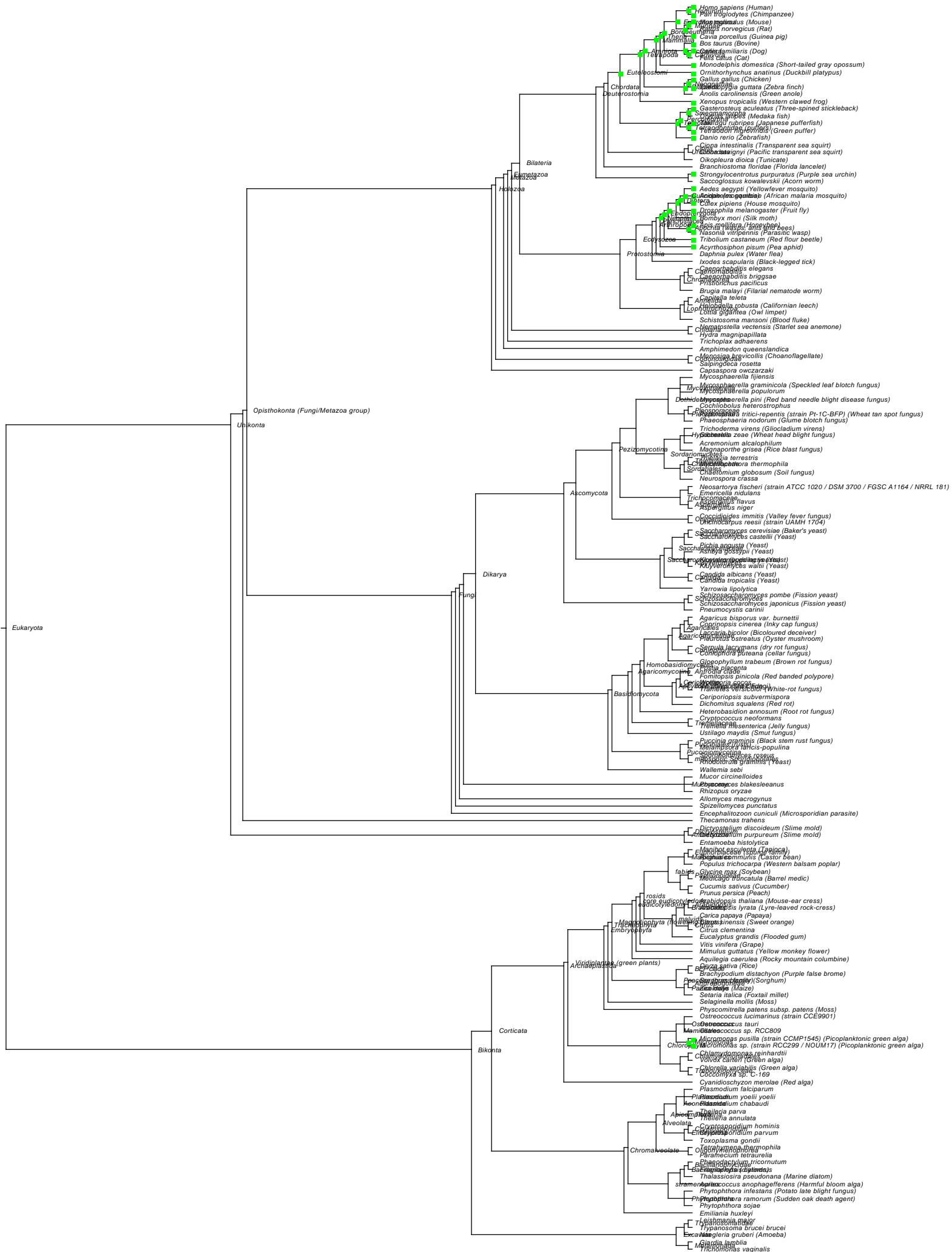

Supplement: Figure S6 — Parallel evolution of the K Homology (KH)∼DEAD/DEAH box helicase combination between Bilateria and Micromonas . Tree nodes in which the domain combination in question has been inferred to be present are highlighted in green. Summary of conditions used: protein predictions as listed in Table S1, domain models from Pfam 25.0, analyzed with HMMER 3.0, Pfam “gathering” cutoffs. (PDF) [file pcbi.1002701.s006.pdf]
